# Morning vs. evening growth hormone injections and their impact on sleep-wake patterns and daytime alertness

**DOI:** 10.3389/fendo.2025.1483199

**Published:** 2025-02-17

**Authors:** Aglaya Levshtein, Mohamad Sharkia, Maya Shimshi-Barash, Tal Almagor, Kerstin Albertsson-Wikland, Zeev Hochberg, Giora Pillar, Alina German

**Affiliations:** ^1^ Pediatric Endocrinology and Diabetes Unit, HaEmek Medical Center, Afula, Israel; ^2^ Pediatric Department, Carmel Medical Center, Haifa, Israel; ^3^ Faculty of Medicine, Technion—Israel Institute of Technology, Haifa, Israel; ^4^ Institute of Clinical Sciences, Dept Pediatrics, Sahlgrenska Academy, Gothenburg University, Gothenburg, Sweden

**Keywords:** growth hormone therapy, sleep-wake patterns, daytime alertness, IGF-1 level, actigraphy, crossover trial, morning growth hormone injections, evening growth hormone injections

## Abstract

**Context:**

Physiological growth hormone is secreted during slow-wave sleep. Traditionally, growth hormone (GH) therapy is given in daily GH injections before sleep. While morning and evening GH injections produce comparable effects on growth and IGF-1 levels, the evening schedule better imitates the physiological diurnal pattern of GH secretion and action. However, the inflexibility of bedtime injection schedules, coupled with the discomfort and psychological distress associated with the injection and local reaction, may cause sleep disturbances in patients, and may significantly burden them and their families.

**Objective:**

Our objective was to evaluate evening vs. morning daily GH injections with respect to sleep-wake pattern, duration, and activity index in children treated with growth hormone.

**Design:**

An open-label, randomized crossover trial of 20 children (11 boys) 5–14 years of age with isolated growth hormone deficiency (n=12) and idiopathic short stature (n=8) treated with daily injections of median GH dose 33 (range13-46) mcg/kg/d was performed. Each subject received 2 weeks of evening injections and 2 weeks of morning injections. Patients' sleep-wake patterns and activity index were assessed by a 7-day actigraph covering the second week of each treatment schedule.

**Results:**

All subjects slept well, within recommended ranges for sleep parameters, regardless of whether they were receiving morning or evening GH injections. Results were comparable for all measures: total time in bed (min), 526.0 ± 51.8 vs 516.9 ± 57.4 for evening and morning GH injections, respectively; total sleep time (min), 512.4 ± 51.1 vs 504.3 ± 57.7; sleep efficiency (%), 93.6 ± 2.6 vs 94.2 ± 2.3; sleep onset latency (min), 8.9 ± 8.1 vs 7.4 ± 6.8; number of arousals per night, 14.5 ± 5.4 vs 12.5 ± 5.2; and 24-hour activity index, 68.3 ± 4.0 vs 67.0 ± 5.0, respectively. No difference was found between the growth hormone deficient and idiopathic short stature group. No difference was found between boys and girls.

**Conclusions:**

Sleep-wake patterns and activity index were not affected by treatment schedules. We recommend that growth hormone injections take place at any regular time according to the family's convenience.

## 1 Introduction

The goal in growth-promoting therapy in children with growth disorders is to increase the child's growth rate and adult height while approaching a physiological pattern of growth hormone (GH) secretion. Treatment is given by daily subcutaneous injections, traditionally administered at bedtime (1, 2), through childhood and adolescence until the child reaches adult height. Evening GH injection better imitates the physiological diurnal pattern of GH secretion (3) and is supposed to improve sleep in growth hormone deficiency (GHD) patients (4). However, lack of flexibility in the treatment schedule may have a negative effect on compliance. Specifically, injection-related pain and bruising are prevalent concerns among both children and their caregivers, with additional emotional burdens including injection-related fear, anxiety, and stigma further complicating treatment adherence (5).

The connections between sleep patterns and growth, learning, attention, and behavioral function are well-established (6–8). In children, insufficient sleep quantity or quality and consequent daytime sleepiness may lead to poorer emotion regulation, a shorter attention span (9), lower school performance (10), and an increased risk of overweight and diabetes later in life (9, 11, 12). The traditional evening GH injection schedule is intended to improve sleep in GHD patients. Recent studies have identified fear, stressful conditions, and discomfort among factors that both children (aged 9–12 years) and their parents perceive as contributing to inadequate sleep (12). As such, the discomfort and psychological distress associated with the injection routine and the local reaction, coupled with the inflexibility of bedtime injection schedules, may cause sleep disturbances in patients, and may significantly burden them and their families.

In addition, there are physiological reasons to question the efficacy of the evening schedule in improving sleep. The interplay between the somatotropic axis and sleep regulation has been extensively documented in both preclinical and clinical studies, demonstrating the integral role of growth hormone–releasing hormone (GHRH), somatostatin, GH itself, insulin-like growth factor-I (IGF-I), synthetic GH secretagogues, and ghrelin in modulating sleep architecture (13, 14). In normal physiological conditions, plasma GH levels are characterized by acute short surges of spontaneous GH secretion. The maximal GH secretory burst occurs within minutes after the first period of slow-wave sleep (SWS) in the first part of the night (13–15). Yet Zadik et al. (16) found that the number of GH pulses during the night did not differ significantly between normal children and slow-growing subjects with deficient 24-h GH secretion. Rather, they found that the differences in integrated 24-h concentration of GH between normally growing and poorly growing children are due to a lower amplitude of peaks during the daytime hours. They hypothesized that the numbers of GH peaks is the last thing lost when there is a GH secretory abnormality (16).

In normal individuals, sleep-onset GH secretion appears to be primarily regulated by GHRH stimulation during a period of relative somatostatin withdrawal. In humans, GH secretion during early sleep may be nearly totally suppressed by administration of a specific GHRH antagonist (17). In most studies, both the duration of SWS and the intensity of slow-wave activity are increased by GHRH, and use of GHRH antagonists decreases both non-REM sleep and GH secretion (18). Stimulation of nocturnal GH release and stimulation of SWS are thought to reflect synchronous activity of at least two populations of hypothalamic GHRH neurons (17). It is therefore likely that stimulation of the GH axis and of SWS production are not only synchronous events but are also closely interrelated (17). Indeed, GHRH represents an important sleep-promoting substance, and in keeping, has been found to be essentially controlled by sleep–wake homeostasis (19). When the sleep cycle is delayed or advanced, GH secretion is consequently delayed or advanced to coincide with the first episode of sleep (20, 21).

Growth hormone injections are traditionally timed to align with the natural circadian rhythms of GH secretion, typically during the early night, to coincide with the onset of slow-wave sleep (22). However, exogenous GH injections, serving as a replacement for endogenous GH, lead to the inhibition of endogenous GHRH secretion. In most children, exogenous GH remains in the serum for 10–16 hours following injection (23, 24), during which time it inhibits endogenous GHRH secretion, potentially leading to impaired sleep quality.

We hypothesized that daily GH injections given in the evening could disrupt sleep both by inhibiting GHRH secretion, and due to the discomfort and psychological distress associated with the injection and local reactions. By contrast, morning injections should have the opposite effect by enabling physiological GHRH secretion before sleep, generating a sleep-promoting effect and enabling pulses of endogenous GH secretion during the night.

Actigraphy involves a portable device, typically worn on the wrist, which records movement over extended periods to estimate sleep-wake cycles via motion detection. Actigraphy offers a non-invasive and practical approach for the longitudinal monitoring of sleep patterns in a home environment (25, 26).

The present study was designed to evaluate evening vs. morning administration of a GH injections with respect to sleep quality, duration, and daytime activity in an open-label, randomized, crossover design in children with GHD or idiopathic short stature (ISS). Sleep was evaluated via a sleep log and a wrist actigraph, which was shown to provide a reasonably accurate estimation of sleep and wakefulness (4).

## 2 Materials and methods

### 2.1 Study design

**Figure 1:**
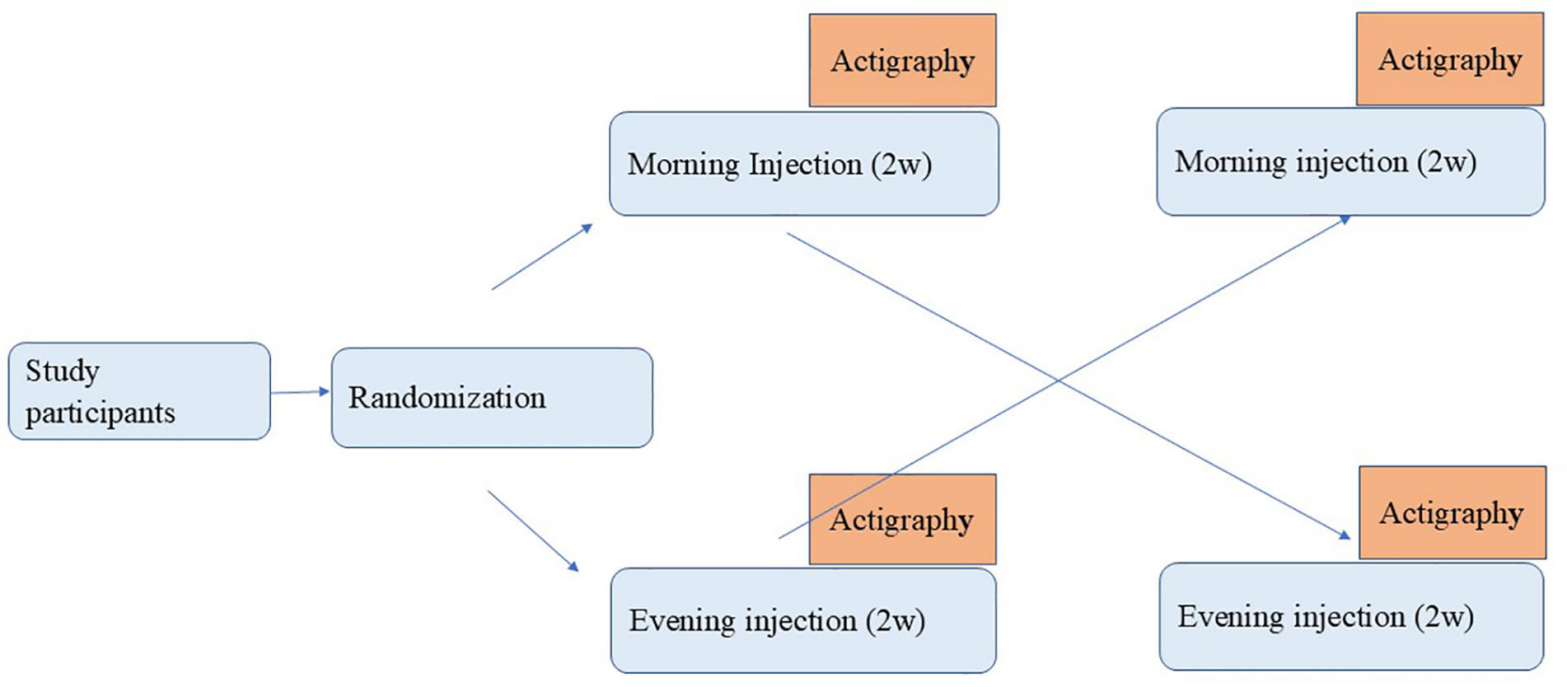
Study design.

This was an open-label, randomized crossover trial, designed to compare sleep-wake pattern and daytime activity with a morning or evening GH dose in children treated with recombinant human growth hormone. Patients received standard GH therapy with a subcutaneous recombinant growth hormone injection (Genotropin, Pfizer, USA, or Norditropin, Novo Nordisk, Denmark). The study lasted four weeks, with each patient receiving their daily GH in the morning for two weeks and at bedtime for the other two (**Figure 1**). Patients were randomized into two groups, with half receiving a morning dose as their initial treatment schedule, and half an evening dose. Randomization was performed to assign the initial schedule of injection timing morning or evening for each participant. This random assignment ensured that half of the participants started the study with morning injections and the other half with evening injections.The schedules were standardized to 08:00–09:00 and 20:00–21:00, respectively, according to the patient's age. Patients received standard therapy with a GH dose identical to each subject's prestudy therapy, with median 33 (range 13–46) mcg/kg/d. The protocol was approved by the HaEmek Medical Center Ethics committee and conducted in accordance with the Declaration of Helsinki. Parents of all subjects provided written informed consent.

### 2.2 Patients

The study cohort were twenty children treated for (11 males and 9 females), median age 10.5 years, range 6–14 years, who were on follow-up at the Pediatric Endocrinology Clinic at HaEmek Medical Center in 2015–2022 and agreed to participate the study. Most patients, 17 of 20, were prepubertal, one girl with GHD and two girls with ISS had breast development Tanner stage 2. All GHRH tests were performed in the same laboratory.

Based on clonidine and/or arginine growth hormone stimulation tests, 12 patients were diagnosed with GHD (peak GH below 7.5 ng/ml) and 8 with idiopathic short stature (peak GH above 7.5 ng/ml). Baseline GH values were obtained by averaging the values obtained 30 min before and at the time of clonidine and/or arginine injection. The maximum GH response was defined as the increment above the baseline value. All GHRH tests were begun between the hours of 07:00 and 09:00. In all patients with GHD, brain imaging was normal.

At the time of the study, all patients had been receiving a daily injection of recombinant human growth hormone (Genotropin, Pfizer, or Norditropin, Novo Nordisk) in the evening for at least 6 months. Those enrolled in the study all had reasonable control determined by mean insulin growth factor (IGF-1) levels in the previous year (median -0.3, range -2.7–2.2 ng/ml), with no differences between the GHD and ISS patients. Exclusion criteria included prematurity, being small for gestational age, neurological or psychiatric disorders, ADHD, movement disorders, syndromic short stature, additional pituitary hormone deficiency, known sleep disorder, known medical therapy for a chronic condition, or less than 6 months of growth hormone treatment. Two patients with idiopathic short stature were excluded during the study due to poor compliance. The 20 patients described here comprise the final sample for analysis after those exclusions. Patients' clinical characteristics are presented in **Table 1**.

**Table 1:** Clinical parameters of all study participants.

Patient characteristics	All patients	GHD n=12	ISS n=8	p-value
No. of patients (males/females)	20 (11/9)	12 (9/3)	8 (2/6)	0.08
Age (years)	10.5 (5–14)	10.5 (6–14)	10.5 (5–14)	0.94
Weight (SDS)	-0.75 (-3;+2)	-0.46 (-3;+2)	-0.85 (-3;0)	0.22
Height (SDS) at study entry for chronological age	-1.0 (-3.1;+1.6)	-0.80 (-2.7;+1.6)	-1.35 (-3.1;-0.6)	0.15
BMI (SDS)	0.00 (-1.9;+1.5)	0.10 (-1.9;+1.5)	-0.17 (-1.7;+0.8)	0.43
GH dosage (mcg\kg), median (range)	33 (13–46)	32 (13–40)	35 (30–46)	0.14
Puberty (yes/no)	3/17	1/11	2/6	
IGF-1 median (range) for age ng/ml	-0.3 (-2.7; 2.2)	-0.2 (-2.7; -1.5)	-0.3 (-1.7;2.2)	
MRI normal/abnormal		12/0		

Data are presented as median (range).

### 2.3 Evaluation

Reference values were obtained from Dahl (7). Sleep and daytime activity were assessed by a 7-d actigraph which monitored total sleep time, number of arousals, sleep efficiency, sleep latency, and daytime movements (see section 2.4). The actigraph was worn during the second week of each treatment schedule (27). A sleep log was used to capture time of lights off in the evening and lights on in the morning.

### 2.4 Actigraphy

Continuous actigraph monitoring was used to identify sleep-wake patterns and daytime activity. The actigraph (Individual Monitoring Systems, Inc., Baltimore, MD) is a small watch-like device weighing 10 g that records movement accelerations. It is worn all day and night (24 h, except when bathing or showering) on the wrist of the nondominant hand. The actigraph uses a validated algorithm to translate the presence of movement accelerations into the variables of interest (9, 10). Actigraph data were processed and scored for the following variables: time in bed (time from lights off until lights on); total sleep time (actual time the child slept); total wake time after sleep onset (time spent awake between initial falling asleep and lights on); sleep onset latency (time from reported lights off until initial falling asleep); sleep efficiency (total sleep time as a percentage of time in bed); number of arousals; and activity index (intensity of movement acceleration during a given time). The accepted definition of sleep disturbances on the basis of actigraphic sleep/awake study is of sleep efficiency of less then 90% and/or three or more awakenings on a nightly average (28).

### 2.5 Statistical analyses

The primary outcomes studied were changes in the activity index and number of arousals per night. The remaining variables—time in bed, total sleep time, total wake time, sleep onset latency, and sleep efficiency—were secondary outcome measures. The sample size was selected to allow for 80% power to detect a difference of 20 in U-index activity and two nocturnal arousals per night between the two groups with SD at an a-level of 0.05. Based on our experience with actigraphy, the standard deviation in activity in normal children is ± 20 in index activity and ± 2 arousals per night. Thus, we chose these numbers for sample size calculations, to discriminate the groups by at least 1 STD in each measure. Sleep parameters were compared using the paired t-test. Therefore, results for sleep parameters are given as mean ± SD.

## 3 Results

Clinical and auxological parameters were similar between all groups of patients and presented in **Table 1**.

Sleep parameters did not differ significantly between GHD and ISS patients, or between boys and girls (data available on request). In addition, there were no differences in daytime activity indices or in sleep parameters between the evening and morning initial treatment schedules. Subjects who started the study with a morning dose did not differ in their sleep parameters from those who started with an evening dose.

Our main concern was whether the outcome variables would differ between periods when patients received an evening dose, and when they received a morning dose. No such differences were found. Specifically, the number of arousals, total sleep time, sleep efficiency, sleep onset latency, time in bed, and activity indices were comparable between evening and morning injection treatment schedules and were within the normal pediatric range. The results are presented in **Table 2**.

**Table 2:** Sleep parameters and daytime activity with evening vs morning growth hormone injection in all study patients.

Sleep parameters	Evening dose, n=20	Morning dose, n=20	p-value	Normal childhood range
No. arousals/night	14.5 ± 5.4	12.5 ± 5.2	NS	0–6
Total sleep time (min/night)	512.4 ± 51.1	504.3 ± 57.7	NS	360–600
Sleep efficiency (%)	93.6 ± 2.6	94.2 ± 2.3	NS	92–100
Sleep onset latency (min)	8.9 ± 8.1	7.4 ± 6.8	NS	0-20
Time in bed (min)	526.0 ± 51.8	516.9 ± 57.4	NS	
Activity index 24 h	68.3 ± 4.0	67.0 ± 5.0	NS	

No reference ranges were determined for 24-h activity index and time in bed. Activity index = intensity of movement acceleration during a given time; sleep efficiency = total sleep time as a percentage of time in bed; sleep onset latency = time from reported lights off till time of falling asleep; total sleep time = total time the child actually slept. Data are presented as mean ± SD.

## 4 Discussion

We compared morning vs evening GH injection schedules in children with growth disorders (GHD and ISS). The findings show that with respect to sleep-wake pattern, sleep duration, and daytime activity the two schedules were comparable, and all patients slept well within the normal pediatric range. These results indicate that GH therapy can allow for flexible dosing schedules without compromising sleep-wake pattern, potentially improving treatment compliance and patient comfort.

The findings call for rejecting our study hypothesis, which was based on suggestions that the physiological effects and emotional burdens of daily bedtime injections, including injection-related fear, anxiety, and stigma (5), could affect sleep-wake pattern, total sleep time, sleep efficiency, number of arousals, and daytime activity. In fact, none of these parameters differed based on whether the patient was on an evening or morning treatment schedule. Our findings suggest that children may enjoy deep sleep that overcomes an apparent stressful effect of regular evening injections. Indeed, it is well-documented that sleep in children is deeper than in adults. It has even recently been suggested that cortical electroencephalogram arousal patterns are not sufficiently sensitive to detect arousals from sleep in children, and that outputs of the autonomic nervous system may be a better alternative for this purpose (29).

The goal of growth-promoting therapy in children is indeed to enhance growth rates and adult height while attempting to mimic physiological growth hormone (GH) secretion patterns. Considering the pharmacokinetics of human growth hormone (hGH), the common practice of administering subcutaneous human growth hormone (hGH) injections in the evening between 20:00 and 21:00 leads to a peak in hormone levels approximately 4–6 hours later, aligning closely with the natural nocturnal surge that typically occurs around midnight to 02:00 (23). Despite inhibiting endogenous GHRH secretion, exogenous hGH is expected to exert its sleep-promoting effects. This has been observed in GHD patients undergoing GH replacement therapy, where it partially normalizes sleep patterns (14).

Switching to morning GH injections would indeed alter this pattern, causing higher circulating GH levels during daytime hours than under normal physiological conditions. The activity of GH administered in the morning declines around 10–12 hours post-injection (23), potentially increasing evening secretion of growth hormone-releasing hormone (GHRH) and subsequently promoting GH secretion and slow-wave sleep (SWS) during the night (19, 30). Evening injections should be recommended in order to achieve GH patterns that imitates physiology more closely, however, both morning and evening injections deliver constant supraphysiological doses of GH determined by body weight or surface area, based that do not precisely replicate the natural, pulsatile pattern of GH secretion observed in normal individuals and in individuals with partial GH deficiency (24).

Our findings join other research challenging the traditional preference for evening injections, which are intended to mimic the natural nocturnal surge in GH secretion. Zadik et al. examined the effects of administering growth hormone at different times of day in prepubertal children with short stature with normal response to a pharmacological stimulation test and subnormal 24-hour spontaneous GH secretion. Subjects were randomized into morning, afternoon, and evening GH injection groups. After 6 and 12 months, growth rates, IGF-I, and GH-binding protein (GH-BP) levels increased similarly in all groups, indicating that GH timing does not affect treatment efficacy (24). Jørgensen et al. also reported similar IGF-I levels and growth rates between different injection times (31). Likewise, Matustik et al. and Chennaoui et al. found no differences in IGF-1 patterns or 4-month growth response when comparing morning and evening GH injections in children with GH deficiency (32) and short normal children (33).

Exploring whether metabolic effect of growth hormone is equally beneficial with morning versus evening growth hormone (GH) injections in GH-deficient patients is crucial for tailoring optimal treatment plans for metabolic stability and overall health. Few studies have addressed the topic. Evidence indicates that evening GH injections may better support fat metabolism and protein conservation due to their synchronization with natural hormonal cycles. Research by Jorgensen et al. reveals that evening GH injections enhance the availability of GH over 12 hours and significantly increase non-esterified fatty acid (NEFA) levels (31), boosting lipolysis while potentially causing increased insulin resistance and hepatic glucose production due to NEFA's antagonistic effects on insulin, especially as evening insulin sensitivity naturally wanes (34–37). Conversely, morning GH injections raise daytime insulin (31) levels likely due to GH's counterregulatory actions on insulin's effects, with a less pronounced impact on insulin sensitivity and glucose levels attributed to naturally higher morning insulin sensitivity. To better understand and optimize GH therapy for GH-deficient patients, further studies should focus on comparing the long-term metabolic impacts, of morning versus evening GH injections, particularly evaluating how they affect lipolysis, protein conservation, and glucose regulation, to enhance overall metabolic health.

The current study's main strength is its randomized crossover design. The study also has a number of potential limitations. First, by focusing on sleep patterns and daytime activity as primary endpoints, the study required only a modest sample size. Second, the short-term nature of the study, with two weeks devoted to each dosing schedule for each patient, allows for examination of short-term parameters, such as sleep and wakefulness, but not longer-term outcomes such as IGF-I levels, growth rates, or bone maturation. Researchers should continue to examine the impact of alternative treatment schedules on such outcomes in children treated with GH. The study cohort consisted of children diagnosed with either isolated GHD or idiopathic short stature. Zadik et al. found no significant differences in GH pulse frequency across a 24-hour period between groups with normal GH secretion and those with isolated GHD, noting between eight and eleven GH pulses in both groups. Differences in GH levels were attributed to peak amplitude rather than pulse frequency. However, in children with organic GHD, no GH peaks were detectable over the 24-hour period (16, 38). Consequently, the study's findings are not directly applicable to children with severe GHD.

The evaluation of sleep in children and adolescents employs a variety of methodologies, each with distinct applications and levels of invasiveness. Polysomnography (PSG) remains the cornerstone for comprehensive sleep assessment, providing detailed insights into sleep stages, respiratory parameters, and neurological activity. As the gold standard, PSG is particularly indispensable in diagnosing sleep-related breathing disorders and complex sleep disturbances.

Actigraphy involves a portable device, typically worn on the wrist, which records movement over extended periods to estimate sleep-wake cycles via motion detection. This technique is essential for examining sleep-wake cycles and circadian rhythms, aiding in the evaluation of daytime sleepiness, and enabling long-term tracking of sleep patterns within a home setting across various groups, such as infants, children, and adolescents (25, 26, 39).

Meltzer et al. in a comprehensive review encompassing 228 studies utilizing actigraphy, showed that this method has been extensively applied to evaluate sleep-wake patterns across various pediatric age groups demonstrating its widespread application across various conditions including developmental disorders and chronic illnesses. The authors demonstrated a consistent finding of high sensitivity but low specificity in actigraphy's ability to detect wake periods after sleep onset. This suggests that while actigraphy is reliable for identifying sleep, it is less so for detecting wakefulness. The authors highlight actigraphy's value in long-term, non-invasive monitoring of sleep in naturalistic settings, though they also point to the need for improved specificity in wake detection (40).

Morning GH injections may complicate children's already hectic routines, increasing stress and anxiety about the pain associated with these injections. The rushed environment of morning preparations for school could heighten children's perception of pain, potentially affecting their cooperation and mood adversely. Evening injections, while potentially worrying due to concerns about affecting sleep, do not seem to disrupt children's sleep patterns according to study data. The calmer evening setting may help children cope better with the anxiety and discomfort from injections, aided by parental support. The timing of GH injections, whether in the morning or evening, does not appear to influence children's daily activities. This suggests that with a consistent routine, GH therapy can be effectively managed without disrupting the child's daily life or activity level.

In conclusion, nighttime and morning hGH administration were found to be equivalent with respect to sleep patterns and daytime activity. In accordance with previous research showing comparable effects of morning and evening administration on growth rates and IGF-1 levels in GHD and short normal children, we find there is no advantage in giving hGH injections in the evening. From a practical perspective, this combined set of findings suggests that allowing flexibility in the timing of GH injections is a viable approach to GH therapy that prioritizes patient-centered care, potentially improving treatment adherence and outcomes without compromising sleep health or growth metrics in children with growth disorders. Future research should aim to validate our findings in larger and more diverse pediatric cohorts, and to explore potential differences in subgroups with varying severity of GHD or higher levels of injection-related anxiety. Such research should be extended over a longer duration to explore how different timing of growth hormone injections influences growth rates, bone maturation and metabolic effects. Additionally, employing more detailed methods of sleep assessment, such as polysomnography, could offer more nuanced insights into subtle alterations in sleep stages not detectable by actigraphy alone.

## Data Availability

The raw data supporting the conclusions of this article will be made available by the authors, without undue reservation.
